# 3D Contractile and Remodeling Behaviors of Functionally Normal and Prolapsed Human Mitral Valve Interstitial Cells

**DOI:** 10.1007/s10439-026-04319-y

**Published:** 2026-08-03

**Authors:** Toni M. West, Gabriel Peery, Sanjana S. Chemuturi, Jodie H. Pham, Giovanni Ferrari, Micheal S. Sacks

**Affiliations:** 1 James T. Willerson Center for Cardiovascular Modeling and Simulation, Oden Institute for Computational Engineering and Sciences, The University of Texas at Austin, 201 E. 24th Street, Austin, TX 78712, USA; 2 Department of Surgery, Columbia University, 177 Fort Washington Avenue, New York, NY 10032, USA

**Keywords:** Extracellular matrix remodeling, 3D traction force microscopy, Mitral valve prolapse, Inverse modeling, Mitral valve interstitial cells

## Abstract

**Purpose:**

Mitral valve prolapse (MVP) can lead to heart failure, arrhythmia, and death. Current treatments for MVP are strictly surgical, while alternative therapies remain elusive due to lack of knowledge of underlying processes. Importantly, our understanding of cellular mechanisms of post-MVP repair remodeling that can lead to secondary surgery remain limited. We therefore explored how MVP affects human mitral valve interstitial cell (MVIC) extracellular matrix (ECM) remodeling and basal contractility.

**Methods:**

Isolated MVP and physiologically normal MVICs were embedded in poly(ethylene) glycol-based hydrogels containing fluorescent microbeads (~ 1 μm) and imaged in the basal and deactivated states. 3D cell surface tractions and changes in MVIC hydrogel local moduli were then determined via inverse computational mechanics modeling.

**Results:**

Hydrogel softening occurred further from MVIC surfaces, whereas pronounced stiffening occurred in close proximity, a result of collagen deposition as verified by collagen staining. MVP MVICs induced greater hydrogel stiffening and less degradation than normal MVICs. Interestingly, even though MVP MVICs had higher basal contractile displacements, their traction forces and hydrogel strain energy densities were significantly lower than those of normal MVICs.

**Conclusion:**

These findings elucidate, for the first time, that MVP MVICs have significantly altered contractile and ECM remodeling behaviors compared to functionally normal MVICs. This result suggests that MVIC behaviors may affect how MVP responds to repair. Moreover, as our studies were performed on isolated MVICs, our observed differences in MVIC behaviors are intrinsic to MVIC phenotype and are not only due to the altered cellular microenvironment.

## Introduction

Non-rheumatic primary mitral valve (MV) disease accounts for approximately 40,000 deaths worldwide each year, with mitral valve prolapse (MVP) representing its most prevalent form [1]. MVP typically induces moderate to severe mitral regurgitation (MR), with its concomitant symptoms and mortality [2]. At present, treatment is limited to surgical repair or replacement, both of which have inherent risks and do not prevent adverse post-operative remodeling that can lead to recurrent MR [2, 3]. A central limitation in developing non-surgical therapies is the incomplete understanding of how cellular remodeling processes drive disease progression and recurrence.

The MV leaflet is composed of a collagen-rich extracellular matrix (ECM) maintained by an embedded population of interstitial cells (MVICs) [4]. In pathological scenarios, MVICs adopt an activated phenotype characterized by elevated intracellular expression of *α*-smooth muscle actin ( *α*SMA) and modulation of the MV extracellular matrix (ECM) remodeling. Such behaviors lead to MV leaflet tissue thickening and stiffening, excess collagen deposition and fragmentation, and increased matrix metalloproteinase (MMP) activity [4]. Although these biochemical and histological alterations are well documented, a key mechanobiological question remains unresolved: How do human MVP MVICs alter their mechanical microenvironment through both contractile traction forces and matrix remodeling, relative to normal MVICs? Specifically, it is unclear how differences in contractility and matrix modification interact spatially to influence local tissue mechanical behavior and adaptations to disease. While we and others have utilized ex vivo tissue preparations to understand MV tissue and cellular behaviors [5-9], it remains very difficult to extract embedded MVIC behaviors due to opacity and local structural complexity of the surrounding tissues.

One approach to address these limitations is the study of isolated cells embedded in gels that emulate some aspects of their native 3D microenvironment, term traction force microscopy (TFM). In TFM, cellular surface tractions are estimated by tracking displacements of fiducial markers embedded within translucent hydrogels [10]. From the resulting displacement field, an inverse finite-element analyses is used to recover cellular traction distributions [11-13]. While developed in 1999 [14], conventional 3D-TFM approaches typically assume that the hydrogel remains mechanically homogeneous. This assumption neglects the effects of cell-induced hydrogel degradation and ECM deposition—processes that are especially relevant in MVP, where both proteolysis and collagen synthesis are elevated. As a result, existing methods may misestimate tractions and fail to capture spatially heterogeneous remodeling. Oberai et al. have addressed this issue in part these limitations by accounting for gel degradation [15, 16].

To more fully address these limitations, including their known highly compressible behaviors, we recently developed a computational framework that incorporates *both* localized hydrogel softening due to enzymatic degradation and stiffening due to matrix deposition into the modeling pipeline [17-19]. This approach enables simultaneous estimation of cellular tractions and cell-induced alterations in gel mechanical properties, all resulting in accurate surface traction distributions. In the present study, as it is known that MVP MVICs produce additional *α*SMA [4], we hypothesized that they would have increased basal contractility and surface tractions. Additionally, as MVP MVICs are known to produce both more collagen and MMPs [4], we expected that both stiffer and more compliant hydrogel regions near the MVP MVICs than normal MVICs would occur. Furthermore, we expected that MVICs would produce larger traction forces near cellular protrusions since most actin networks are seen in these areas of stained VICs [17]. Our results presented herein elucidate a more nuanced story.

## Methods

**Fig. 1:**
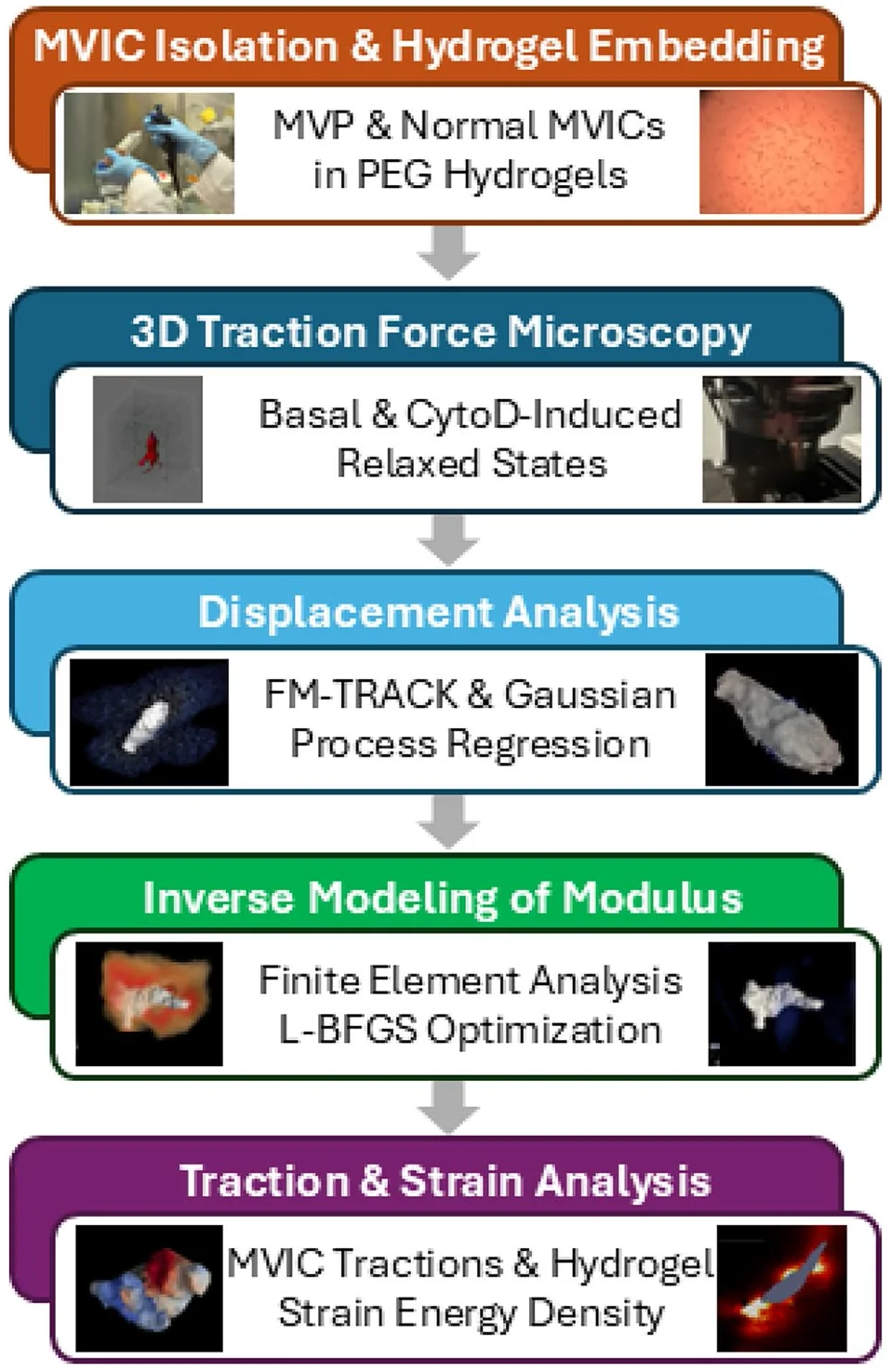
TFM experimental and analysis workflow: overview of the key steps used in the present study to assess the mechanical behavior and ECM remodeling of MVICs from MVP and normal tissue.

MVICs from mitral valve prolapse (MVP) patients and anatomically normal donors (Norm) were embedded in crosslinked poly(ethylene) glycol (PEG)-based hydrogels containing fluorescent-microsphere fiducial markers. These cells were imaged in their basal and fully relaxed states, from which the microsphere marker displacements were determined. The resulting basal MVIC contractility and hydrogel displacement fields were input into a detailed finite-element inverse modeling approach that determined the local hydrogel modulus, using a novel compressible material modeling approach. This approach allowed for accurate recovery of the effects of cellular induced enzymatic degradation and collagen deposition. Moreover, this method then allowed for accurate calculation of MVIC traction forces, as it did not assume that the hydrogel had homogeneous mechanical properties (Fig. 1).

### MVIC Sample Preparation and 3D-TFM Imaging

MVICs were isolated with standard cell culturing procedures as previously described [20] from three patients undergoing first-time surgery for MVP and three anatomically normal (Norm) tissue donors. In brief, the endothelial cell layer was scraped from the leaflets, and the remaining tissue was minced and placed in 1 mg/ml collagenase type 2 solution (Worthington). Cells were then pelleted and resuspended in DMEM + 10% FBS + 1× PSG and grown on plastic to passage p1 to select for interstitial cells and frozen. Expansion was undergone on collagen-coated dishes to prevent non-phenotypic activation of the MVICs [21]. Low passage MVICs, p4–p5, were implemented for hydrogel embedding and imaging to closely preserve the primary cell phenotype, ensuring it remained as representative of native tissue as possible and has been previously described [17, 22]. To summarize, MVICs stained with CellBrite Red were placed in a hydrogel precursor mix containing Dragon green fluorescent microspheres (Bangs laboratories), MMP-degradable crosslinker (Bachem), CRGDS adhesion peptide (Bachem), and norbornene-functionalized 8-arm poly(ethylene) glycol (JenKemUSA) and were gelated with 3 min of UV. The poly(ethylene) glycol-based hydrogels with MMP-degradable crosslinkers were chosen for this study because they provided a controlled microenvironment that preserves key cell behaviors and biomarkers while allowing for high-resolution, 3D measurements of cellular contractility and ECM remodeling, which are not feasible with excised tissue [23, 24]. All materials were obtained from Thermo-Fisher unless otherwise stated. Hydrogels were incubated in DMEM + 10% FBS + 1× PSG for three days prior to imaging. A Nikon AXR laser scanning confocal microscope was used to collect 3D images of MVICs and the surrounding hydrogel fiducial markers in the basal and fully relaxed, cytochalasin-D (CytoD)-treated steady states as previously described [19, 25]. The resulting images had isotropic voxels with side lengths of 0.288 μm and overall volumetric dimensions of 147 × 147 × 119.23 μm after spherical-aberration correction.

### Immunocytochemistry Preparation and Imaging

MVICs were embedded in PEG hydrogels without fiducial markers and fixed with 4% paraformaldehyde in dPBS after three days of culture and stained with wheat germ agglutinin (WGA) conjugated to Alexafluor-555 and rabbit anti-collagen Type 1 antibody (Rockland 600-401-103). Secondary staining was performed with goat anti-rabbit alexafluor-488. Imaging occurred with the same microscope and settings as outlined above.

### Displacement and Volumetric Analysis

Tracking and data analysis of the displacements of the embedded microspheres were performed using our TFM pipeline [19]. First, we tracked the embedded microsphere displacements with our custom developed software FM-TRACK [10] and then interpolated across the hydrogel volume using a Gaussian process regression (GPR) which accounted for noise variance, as described previously [19]. For each MVIC, first-order Lagrangian finite-element meshes were produced for both the MVIC surface (triangular meshes) and the hydrogel volume (tetrahedral meshes). As we assumed the hydrogel and cell were perfectly bonded, the elements between the cell mesh and the hydrogel mesh were coincident. That is, the FMTrack captured displacements of the cellular membrane had a one-to-one nodal correspondence between basal and CytoD states. These displacements were then leveraged to compute the local deformation gradient tensor, F, and the Jacobian, J=det(F). Contractile displacements were defined as those directed toward the cells surface.

### Determination of Spatially Heterogeneous Hydrogel Moduli

As we previously described [19, 22], the hydrogels here were modeled as Neo-Hookean hyperelastic *compressible* solids. The compressibility of the PEG hydrogel has been found to be significant; more importantly, its proper incorporation has been shown to be critical for accurate TFM inverse moduli recovery. We thus used the following spatially varying form for the PEG hydrogel strain energy density

(1) $$\Psi (x_{0})={\bar{c}}_{1}(x_{0})\left(I_{1}-3-2\;\text{ln}\;J\right)+D_{1}{\left(\text{ln}\;J\right)}^{2},$$

where $x_{0}$ is the material point location in the hydrogel, $\Psi (x_{0})$ is the strain energy density, and $I_{1}=\text{tr}\;C$ is the first invariant of the right Cauchy–Green tensor $C=F^{T}F$. The heterogeneous material parameter ${\bar{c}}_{1}(x_{0})$ was given an exponential form:

(2) $${\bar{c}}_{1}(x_{0})=c_{1}e^{\beta (x_{0})},$$

so that large orders of magnitude in modulus could be accounted for with numerical stability by adjusting the $\beta (x_{0})$ term. The material parameter $c_{1}$ was set to one half the mean shear modulus as measured by rheometry, with $c_{1}$ = 108 Pa/2 = 54 Pa, from the small-strain limit of a Neo-Hookean model the shear modulus satisfies $\mu =2c_{1}$. Based on previous results [19], $D_{1}∕c_{1}=1$ was utilized to match the observed compressibility of the hydrogel. Nodal values of $\beta (x_{0})$ encoded a field discretized in the first-order Lagrange basis of the hydrogel mesh. Forward solves were performed in Python FEniCS 2019.1.0 [26-33].

Inverse-model optimization was performed with the limited memory Broyden–Fletcher–Goldfarb–Shanno (L-BFGS) algorithm [34] by matching displacements between the target and simulation starting from initial guess $\beta (x_{0})=0$. The objective function, Φ, that was minimized throughout this process with respect to $\beta_{\text{sim}}$ was

(3) $$\Phi =\int_{\Omega_{\text{EH}}}{\left\|u_{\text{sim}}-u_{\text{tar}}\right\|}^{2}d\Omega +\gamma \int_{\Omega }\nabla \beta_{\text{sim}}\cdot \nabla \beta_{\text{sim}}d\Omega ,$$

where $u_{\text{sim}}$ and $u_{\text{tar}}$ are the simulated and the target displacements, respectively, Ω is the imaged volume, and $\Omega_{\text{EH}}$ is the volume within the “event horizon” defined here as the volume in which microsphere displacement magnitudes could be reliably determined. Regularization is employed both for numerical stability and addressing non-uniqueness of the inverse problem while noise is present in target displacements. Tikhonov regularization penalizes highly oscillatory patterns in $\beta_{\text{sim}}$, moderating the consistent-tangent matrix’s condition number [35]. The final regularization parameter value γ was previously determined [19] by extensive synthetic test cases that utilized a realistic level of noise. The event horizon was defined by a minimum cutoff target displacement magnitude 0.38 μm, chosen to encompass 3 standard deviations of noise in the displacement magnitude as previously described in [19, 22]. By focusing the inverse modeling on $\Omega_{\text{EH}}$, the problem size was tractable using a desktop computer with an i9 processor containing 24 cores and 125 GB allocated RAM.

Gradients of Φ, as required by L-BFGS, were computed with FEniCS-adjoint [36] and MOOLA [37] so that solving this highly-nonlinear inverse problem with large gradients remained numerically stable through spatial weighting of the gradient to promote spatially-smooth guesses, $\beta (x_{0})$, in our adjoint-based gradient computations. In pilot studies, this additional enforcement of smoothness was found necessary in addition to that provided by Tikhonov regularization in the objective in Eq. (3). Simulated displacements at each iteration of inverse optimization were calculated by minimizing total hydrogel strain energy:

(4) $$\Pi =\int_{\Omega }\Psi \;d\Omega .$$

The experimentally observed displacements for each cellular system were imposed on the MVIC surface and hydrogel bounding box through the use of Dirichlet boundary conditions. These boundary conditions therefore prescribed simulated displacements to match the target at both the imaging boundary of the hydrogel and the surface of the cell. The output of the inverse model was the final modulus field prediction, where ${\bar{c}}_{1}(x_{0})=c_{1}e^{\beta (x_{0})}$. Convergence to these values was considered complete when either the objective function value or the magnitude of its gradient fell below values that we previously observed to produce simulated displacements coincident with ground-truth displacements in a pilot study; error analysis determined that only 1.6% of nodes had errors ≥ 0.38 μm and runtime per hydrogel was approximately 1 h [19].

### Post-Processing

As has been previously presented in [19], the surface traction t referenced to the reference (relaxed) configuration (i.e., force per unit reference area) is

(5) $$t=P\cdot n_{0},$$

where $n_{0}$ is the unit surface normal vector in the reference (relaxed) MVIC configuration that points out from the center of the cell and P is the first Piola–Kirchhoff stress tensor. Next, the local hydrogel strain energy densities, Ψ, were determined for each gel element using Eq. (1). Spatial variations in Ψ reflected two major mechanisms: the overall magnitude of the local deformation field $F(x_{0})$ and local value of ${\bar{c}}_{1}$, thus providing a useful comparison of differences in behavior between MVIC phenotypes.

### Statistical Analysis

During statistical analysis, *p* ≤ 0.05 was considered significant. 2–3 MVICs/patient were analyzed, with each type of analysis employing the same MVICs. Specific statistical tests are detailed in figure legends and text.

## Results

### Basal Contractility Displacement

When comparing the average contractile displacements of MVICs from physiologically normal (Norm) and MVP mitral valves (Table 1), an average effect size of 19.10% (1.565–1.266)/1.565) and a *p* < 0.001 indicated that MVP MVICs experienced significantly larger contractile responses. This is in line with previous reports that MVICs have increased *α*SMA expression in MVP models and supports our hypothesis that activated MVP MVICs would have higher contractile displacements [38, 39].

### Spatial Variation of J in ECM-Rich Hydrogels

Building on prior observations of compressibility in related hydrogels [25], the element-wise distribution of J in the PEG-based hydrogels was analyzed (Fig. 3). The PEG-based hydrogels portrayed large volume changes, with 7.226% of mesh elements in all hydrogels having $J(x_{0})\notin [0.95,\;1.05]$. This finding underscores that PEG-based hydrogels undergo greater than 5% volumetric changes and therefore must be modeled as a compressible material.

### Cell-Mediated Extracellular Matrix Remodeling

To determine the extent to which the MVICs had remodeled their surrounding hydrogels by excreting ECM components, the stiffness of the surroundings was modeled with an inverse method that determined heterogeneous Neo-Hookean material parameters. The output moduli of the MVIC-modified hydrogels were compared to collagen immunofluorescent staining of independent MVICs (Fig. 4). The elements of hydrogel that had greater than the global median of stiffening (83 Pa) correlated in pattern and approximate volume with what was seen in the collagen images, giving confidence that the inverse model modulus outputs capture biological processes of ECM modification.

The resulting moduli of each MVIC TFM model were subdivided into zones where greater than median stiffening had occurred (> 82 Pa) and less than median degradation had occurred (< 36 Pa; Fig. 5a). The hydrogel elements of stiffening and degradation were then separately compared between the Norm and MVP groups. The stiffened regions near MVP MVICs possessed significantly larger moduli than these regions near Norm MVICs (Table 1). This supports our hypothesis and is in line with previous reports that state activated MVICs increase deposition of stiff ECM components such as collagen [4]. Surprisingly, the degraded regions of MVP MVIC hydrogels had significantly less degradation than Norm MVIC hydrogels (Table 1). In the degraded regions, the MVP average hydrogel modulus only fell 34.44 Pa over three days of modification (54 Pa unmodified hydrogel—19.66 Pa average = 34.44 Pa), while the Norm hydrogel modulus fell 37.07 Pa. This data depicts that there is an additional 4.870% decrease in modulus in Norm hydrogels over three days of culture compared to that of MVP hydrogels. Therefore, MVP MVICs produce stiffer ECM both due to an increase in stiffening and a parallel decrease in degradation.

Since the initial inspection of the data suggested the stiffened areas were in close proximity to the MVIC surfaces, an analysis was performed where the moduli were placed in 10 bins according to their distance away from the cell, with stiffened moduli and degraded moduli graphed separately. Stiffening trended toward being higher near the cell surface, but distance was not a significant variable for determining modulus (*p* = 0.09257) by two-way ANOVA due to large variations in stiffened moduli (Fig. 5b; Table 5 in Supplementary Material). Even so, significant differences in moduli could be seen between the differing distances. Normal MVICs stiffened their surroundings approximately 2000 times the unaltered hydrogel modulus within the first 20 μm, which then significantly increased to approximately 20,000 times the unaltered modulus between 20–30 μm (*p* < 0.001 by Tukey post-test). At 30–40 μm away, hydrogels modified by normal MVICs plummeted in stiffness to approximately 6 times the unmodified hydrogel modulus (*p* = 0.005 by Tukey post-test) and then moved to approximately 2 times the unmodified modulus starting at 40 μm. Alternatively, MVP MVICs only stiffen to the 2000× level for the first 10 μm but then continue to stiffen at the more moderate 15× level for 10–50 μm of hydrogel and then drop to about 2 times the unmodified modulus further away.

Degradation with respect to distance from the MVIC was similarly assessed (Fig. 5c; Table 6 in Supplementary Material). The two-way ANOVA with Tukey post-test revealed that degradation stays steady in normal MVICs with distance, causing approximately 40 Pa of change in stiffness from the unaltered hydrogel modulus, no matter the location. In contrast, MVP MVICs have a significant decrease in the amount of degradation with increasing distance from the MVIC, which is a significantly different pattern from the Norm MVICs ( *p* < 0.001 by two-way ANOVA). This highlights that the overall decrease in degradation potential observed in MVP MVICs is primarily due to a decrease in degradation occurring farther away from the cell boundary.

### Cellular Traction Forces

**Fig. 2:**
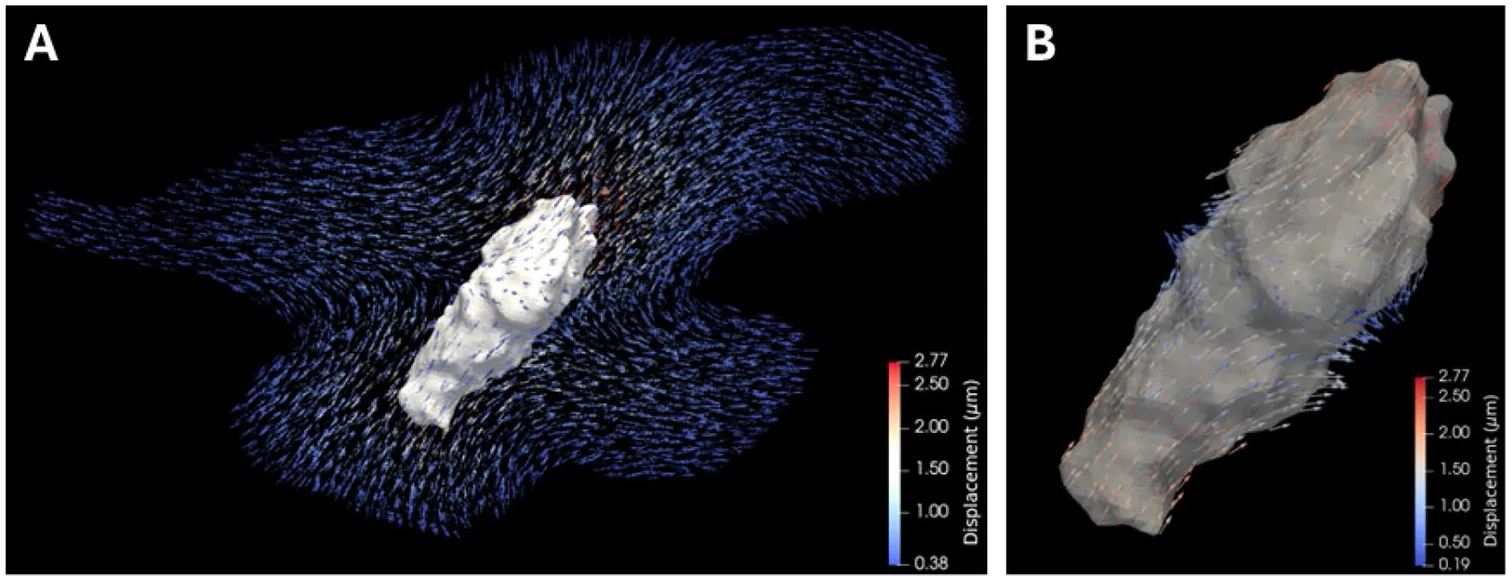
Representative MVIC with basal contractility displacements. **a** Discretization of the displacement field at each hydrogel node within the event horizon of a representative MVIC. **b** Cellular surface displacements of the same representative MVIC

**Fig. 3:**
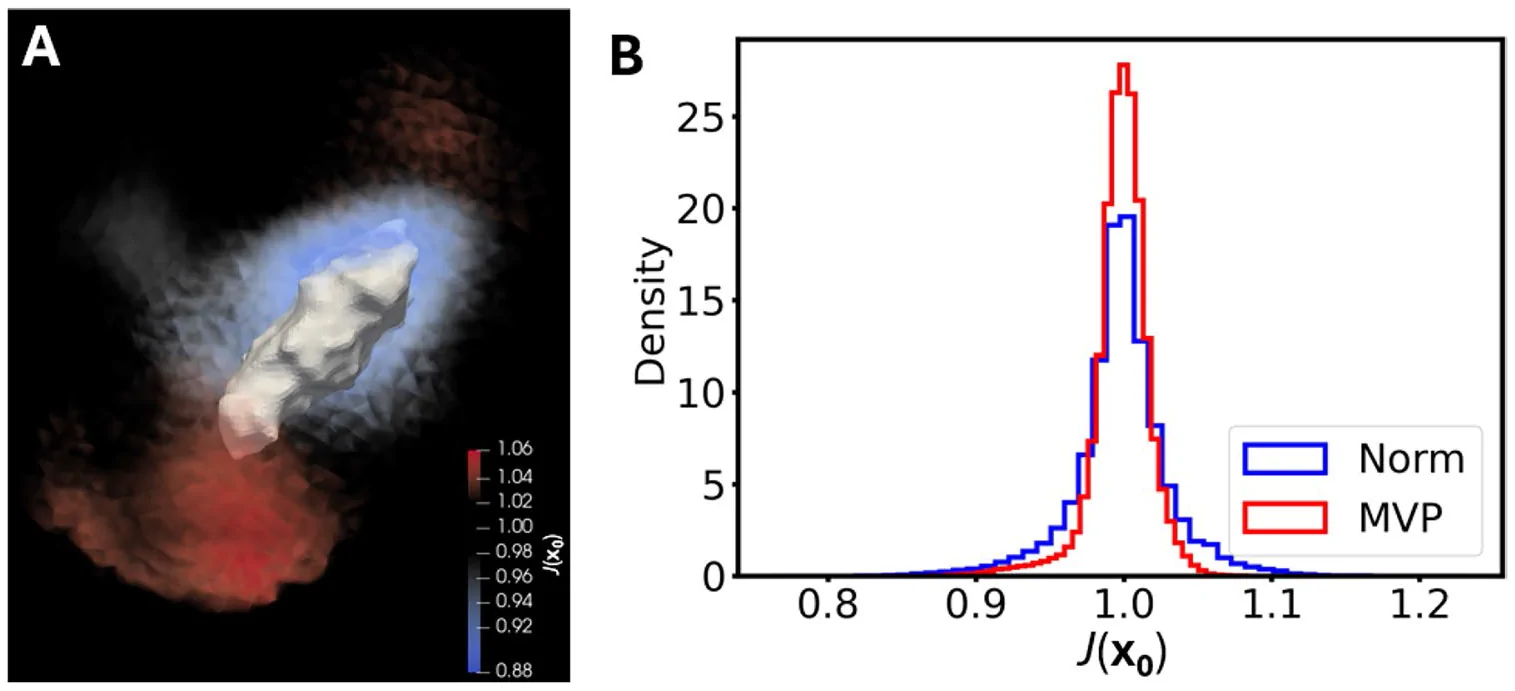
Large variations in J magnitude occur spatially. **a** Rendering of $J(x_{0})$ within the hydrogel for a representative MVIC. **b** Histogram of $J(x_{0})$ from the hydrogels surrounding all MVICs per group. Similarity in both normal and MVP MVIC responses was noted (Earth movers distance test = 0.009), suggesting both cell types can utilize a compressible hydrogel model

**Fig. 4:**
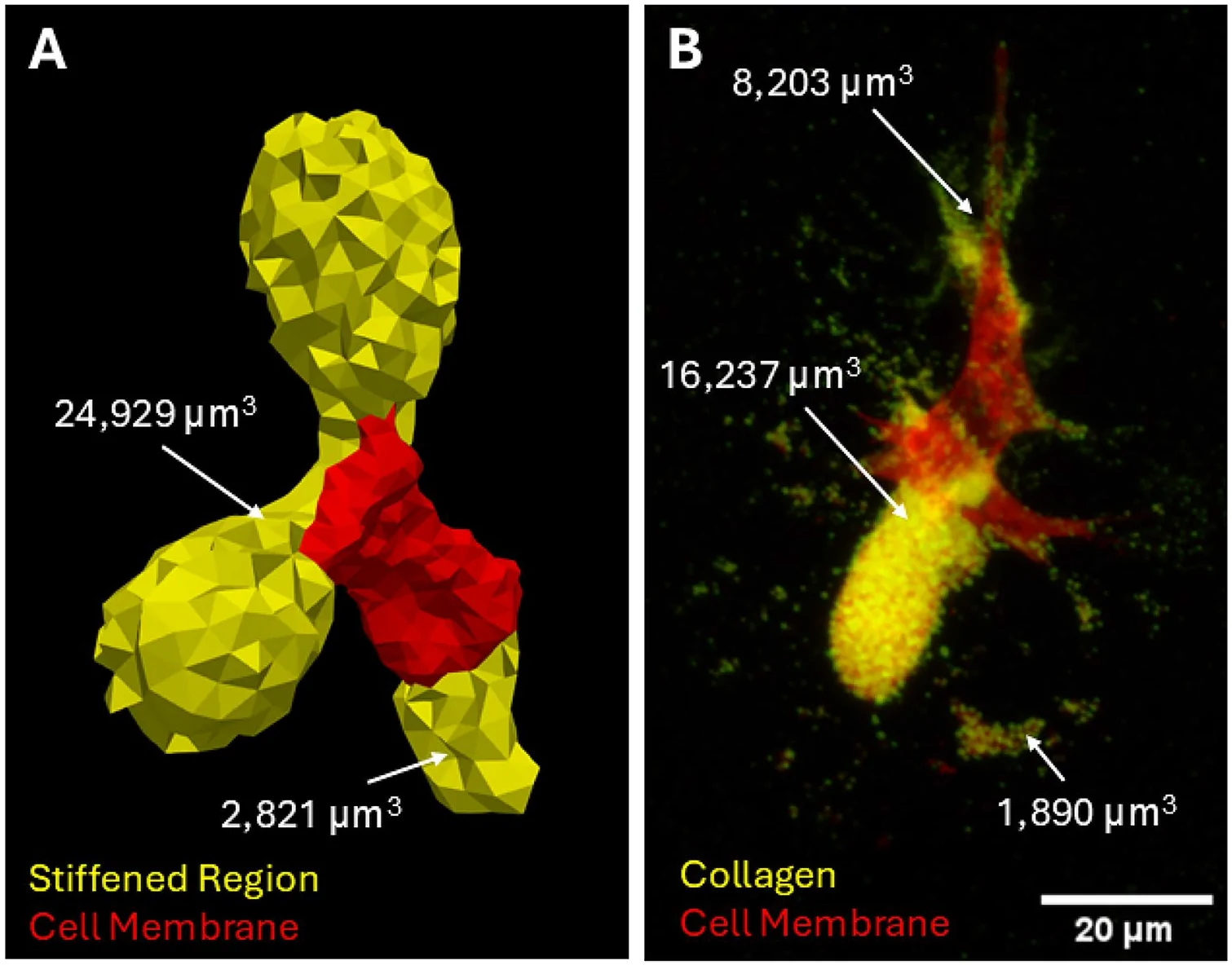
Stiffened hydrogel regions and stained collagen correlate in placement and volume. **a** Rendering of the zones of hydrogel around a representative MVIC that have higher than the global median stiffened value of 82 Pa, as calculated by the inverse model. Total stiffened volume = 27,750 μm^3^. **b** 2D standard deviation projection of a 3D image of an MVIC stained for extracellular collagen. Volumes of individual zones of collagen surrounding the MVIC were analyzed in full 3D in ImageJ-Fiji. Total collagen volume = 26,330 μm^3^. Color legend and scale bar depicted in images.

The surface tractions were found to be significantly lower in MVP MVICs when compared to normal MVICs, suggesting a different mechanism than originally anticipated (Fig. 6; Table 1). Indeed, when the traction-to-displacement ratio (TDR) was calculated, MVP MVICs were approximately 76% less effective at transmitting traction to their surroundings (Norm: 3.193/1.266 = 2.522; MVP: 0.9351/1.565 = 0.5975). Renderings of each MVIC were then reviewed to look for possible trends. When any MVIC displaces during basal contraction, it tends to contract at the protrusions and at the same time have an isovolumetric expansion in the body of the cell (Fig. 2b). Renderings of the MVICs indicated that large traction forces often occurred near protrusions where contraction occurred, so a breakdown of the traction forces by contractile and expansile regions of the MVICs was conducted (Fig. 6; Table 2). In both contractile and expansile regions, MVP MVICs had significantly lower average traction than normal MVICs. Interestingly, there was a switch in the dominance of specific regions. In normal MVICs, larger tractions occur in the expansile regions than in the contractile regions, while in MVP MVICs, larger tractions occur in the contractile regions than the expansile regions, suggesting that cell contractile polarity may be altered during disease progression.

### Spatial Distribution of Strain Energy

To further investigate the effectiveness of how the traction forces from the MVICs affect their surroundings, the strain energy density of the MVIC-modified hydrogel was determined. Strain energy is a particularly attractive biophysical quantity that represents the total energy of cellular contraction, including contractile and gel mechanics. As expected, in all MVICs, strain energy density was highest near the MVIC boundary and significantly decreased with increasing distance from the cell (Fig. 7). Similar to that seen with traction forces, the average of strain energy density from the MVP MVIC ECM was lower than that of the normal MVIC hydrogels (Fig. 7; Table 7 in Supplementary Material), which was mostly due to a decrease in strain energy density 10–30 μm away from the cell membrane in MVP MVIC ECM. But notably, MVP MVICs produced a significantly higher strain energy density within the first 10 μm from the cell boundary. Any difference that occurred between Norm and MVP MVIC hydrogels in strain energy density further than 30 μm away from the cell boundary was not significant and trended toward 0.

**Fig. 5:**
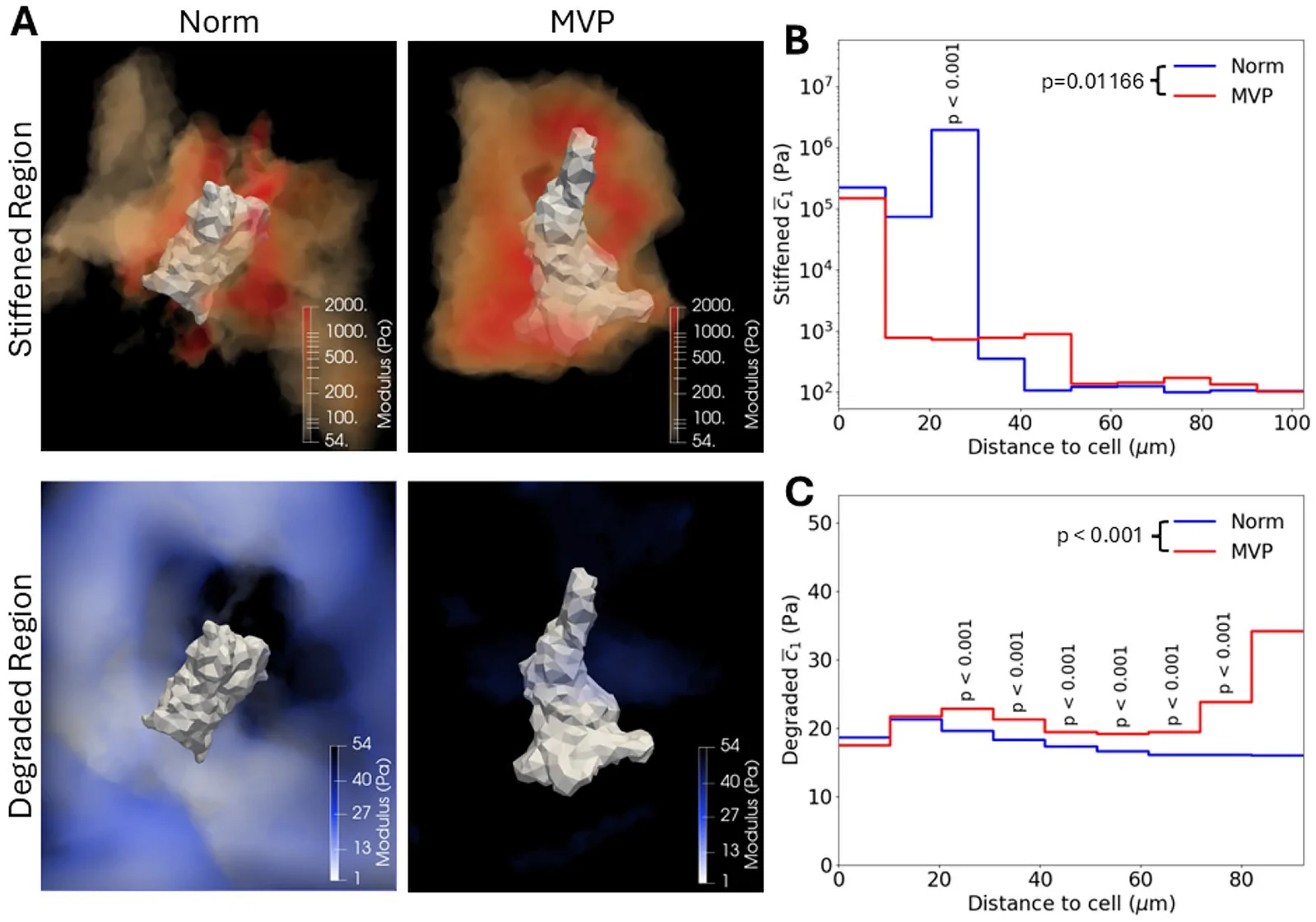
MVP MVICs produce stiffer extracellular matrices. **a** Representative renderings of stiffened and degraded regions of hydrogels modified by MVICs from physiologically normal (Norm) and mitral valve prolapse (MVP) valves. **b–c** Step graphs of stiffened moduli (> 82 Pa; **b**) or degraded moduli (< 36 Pa; **c**) with respect to distance bins of 10 μm each. *p* values on legend calculated by two-way ANOVA. *p* values above steps calculated by Tukey post-test between Norm and MVP groups at that step.

**Fig. 6:**
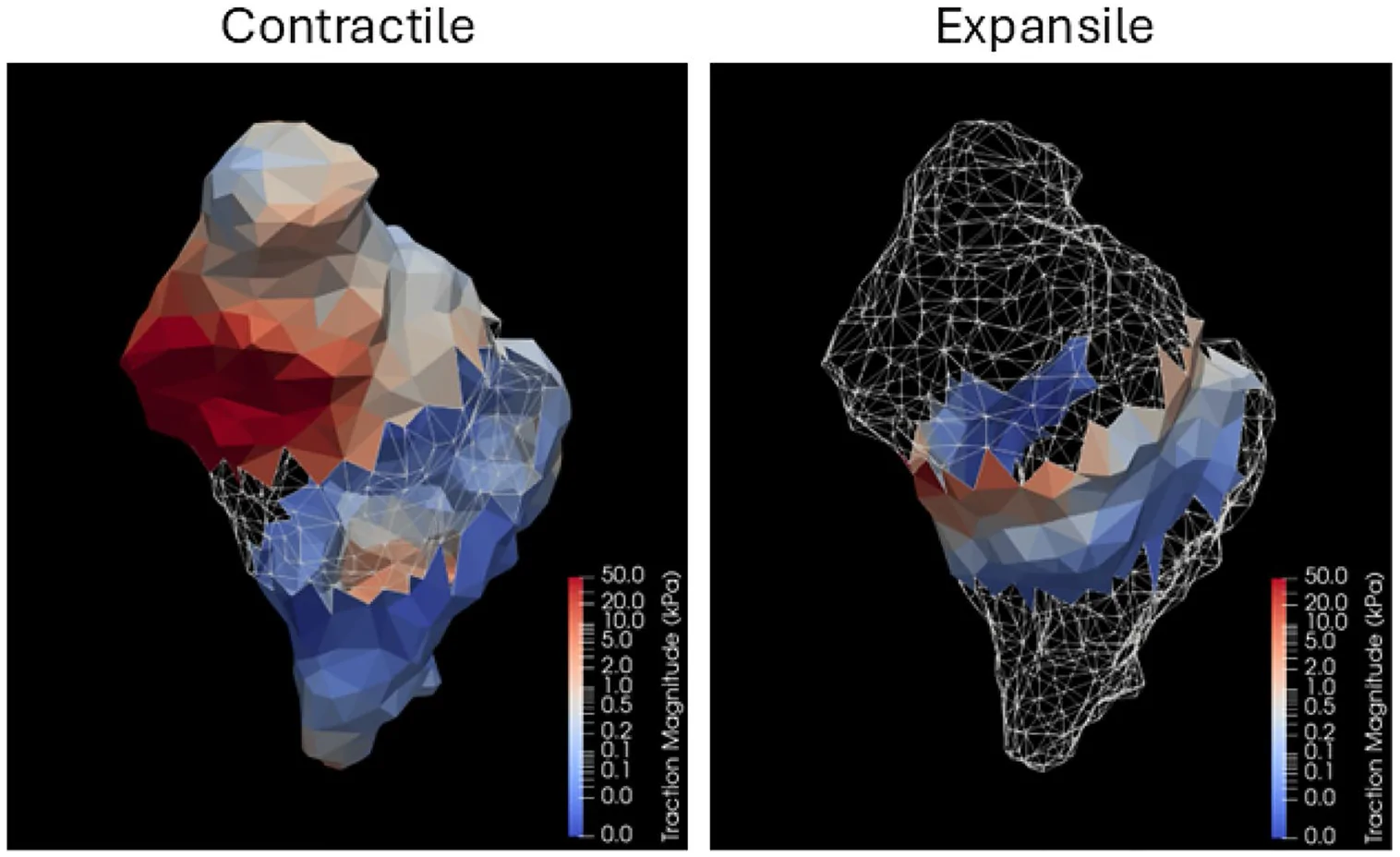
Traction forces in the contractile and expansile regions of a representative MVIC. Rendering of traction forces present on each surface element of an example MVIC depicting the elements where the cell was contracting inward toward the MVIC centroid (Contractile) or expanding outward from the centroid (Expansile)

**Fig. 7:**
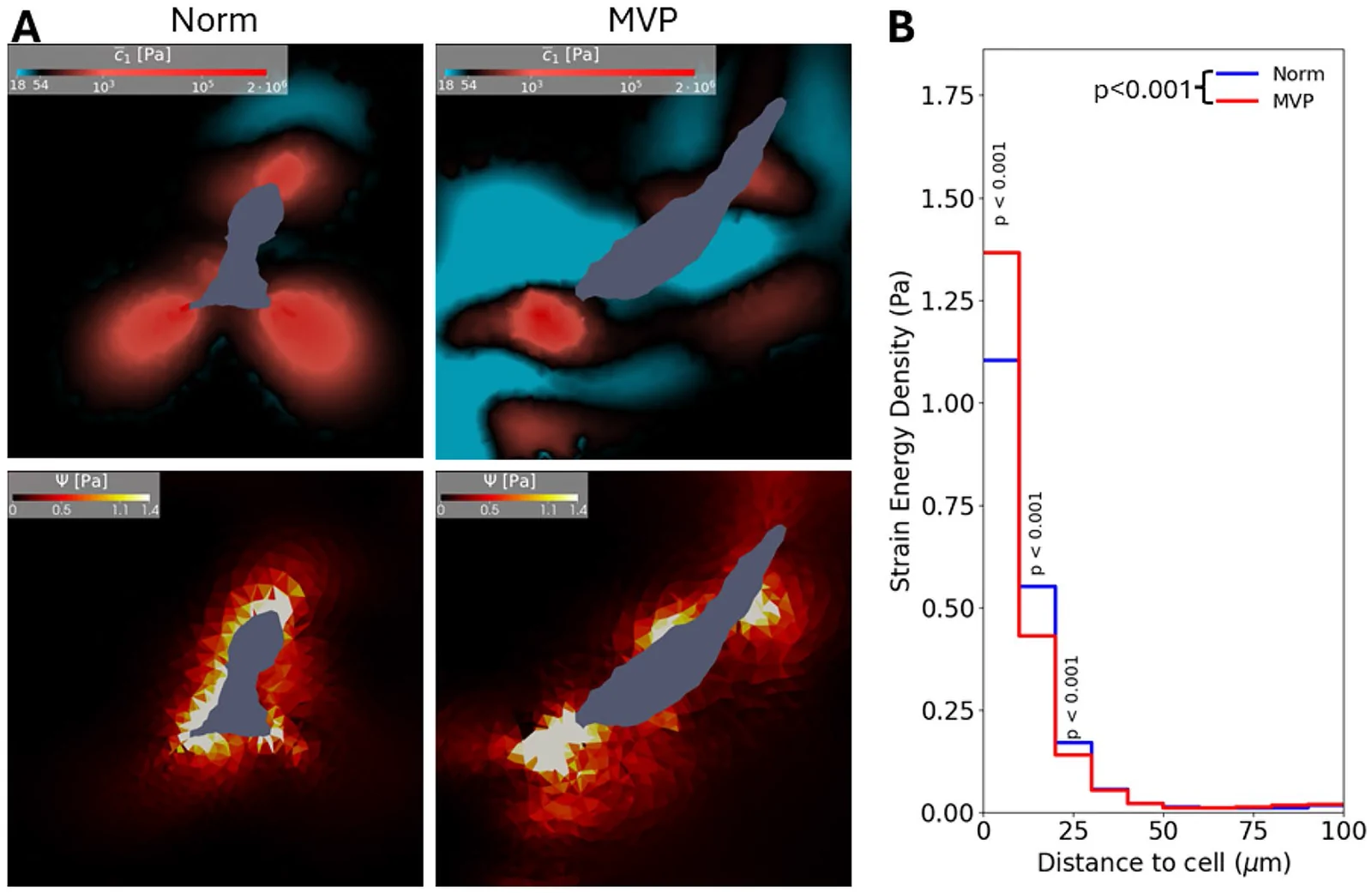
Strain energy density decreases when distance from the MVIC increases, which correlates with regions of high stiffness. **a** Cross-section slices of a normal (Norm) and MVP MVIC where the stiffened moduli (${\bar{c}}_{1}$) and high strain energy density (Ψ) align. **b** Step graph of strain energy density vs binned distances from the cell boundary. *p* value depicted in legend determined by two-way ANOVA. *p* values depicted above steps determined by Tukey post-test.

Also, stiffened areas of ECM had significantly higher strain energy densities than degraded regions (Table 3). To understand this finding, a closer look at the displacement fields (Fig. 2) in relationship to the stiffness fields (Fig. 5) reveals that while there are large gradients in stiffness, the displacement field has much more gradual gradients. So in areas where the stiffness quickly changes from degraded to stiffened, there are not large changes in the cell-mediated hydrogel displacements, meaning that the cell must pull on the stiffened areas of the hydrogel with more energy than the degraded zones to cause the same amount of displacement. These results therefore support our hypothesis that stiffened regions would be placed under higher strain energy density than degraded areas.

Since contractile polarity of cell surface traction forces was altered in disease, an investigation of whether this polarity shift was translated to strain energy density of the surroundings was then conducted. Both hydrogels modified by normal and MVP MVICs exhibited significantly higher strain energy density in contractile regions than in expansile regions. Furthermore, stiffened MVP MVIC ECM exhibited significantly lower strain energy than Norm MVIC ECM, suggesting that the changes in overall strain energy density throughout the MVP ECM are mostly governed by changes that occur in the stiffened regions (Table 4).

## Discussion

### Summary of Major Findings

In the present study, we hypothesized that as MVP MVICs have increased basal contractility, ECM remodeling, and basal traction forces. We found that MVICs isolated from MVP patients produced significantly stiffened hydrogels, whose stiffening was more far-reaching from the cell. Although MVP MVICs had significantly larger basal contractile displacements than Norm MVICs, MVP MVICs produced significantly lower traction forces at the cellular surface and less strain energy density within the hydrogels they modified. Since experimentation occurred on isolated MVICs embedded in PEG hydrogel, the differences in contractility and ECM remodeling that were seen are *intrinsic* to the MVIC cellular phenotype itself. That is, while the micromechanical environment of the MVP MVIC may differ from that of the normal MVIC, the MVP cells demonstrated intrinsic differences.

One aspect of the ECM remodeling that was identified between the MVP and normal groups was that the MVP MVIC hydrogels were less degraded, especially at greater distances from the cell boundary. Since the PEG hydrogel formulation used in this investigation is specifically designed to allow for MMP degradation [22], it was assumed that the difference between MVP and normal hydrogel degradation was due to a difference in MVIC-mediated MMP regulation. Certain secretory MMPs are known to be upregulated during MVP including MMPs 1, 2, 3, 7, and 9 [4], so it was hypothesized that MVP MVIC hydrogels would have higher rates of degradation than hydrogels from normal MVICs. The current data analysis did not support this hypothesis. A review of the literature revealed that because MMPs have relatively small molecular weights compared to other components of ECM or hydrogel matrices, MMPs can move through the matrix over time by Graham’s law of diffusion, but are also affected by the electrochemical and binding interaction between the MMP and the matrix substances [40, 41]. In the MVP MVIC hydrogels, there was a significantly lower amount of degradation further away from the cell. This rise in modulus with distance may be explained by a shift in expression to larger molecular weight secretory MMPs such as MMP-9 (92 kDa) or to MMPs with high affinity to native collagen, like MMP-1. Therefore, future fluorescence correlation spectroscopy experiments looking into the diffusion of MMPs from MVICs with MMP-1 or MMP-9 knockdown may elucidate how MMP expression profile in MVICs plays a role during MVP-related leaflet degradation [41].

The quintessential marker of MVP in MVICs is increased *α*SMA expression [4]. Because this is well substantiated, it was hypothesized that increases of *α*SMA in MVP MVICs would lead to increased cell traction forces and hydrogel strain energy densities. But this was not the case; both basal traction and basal strain energy density were measured to be significantly lower in MVP samples. There are several possible mechanisms that could be occurring to lead to these unexpected results. Firstly, there is not a 1:1 correspondence between *α*SMA expression to its incorporation into polymerized and force generating actin stress fibers. For instance, TGF*β* and ERK signaling up-regulate *α*SMA expression via SMAD and MAPK pathways while *α*SMA incorporation into polymerizing stress fibers is largely mediated by RhoA/ROCK signaling [4, 42]. The latter is more influenced by mechanobiological inputs such as substrate stiffness and intracellular tension than it is by non-canonical TGF*β* and ERK signaling [42] . Therefore, it is possible that although there is increased expression of *α*SMA, there could be a lack of polymerization into actin stress fibers thereafter, which would explain how lower traction forces were seen in MVP MVICs here. In addition, stiffer gel is likely to have a feedback mechanism to alter the formation and force levels of stress fibers within the MVIC.

### Implications

The lack of *α*SMA polymerization may be in part due to the bidirectional feedback loop between focal adhesion stability and stress fiber formation described in fibroblasts, which occurs through ROCK signaling and likely translates to MVICs. Despite being embedded in locally equal or stiffer hydrogel regions, MVP MVICs generated lower cell surface traction forces and exerted less strain energy than normal MVICs, indicating an uncoupling of their adhesion mechanisms. Further investigations into which of these mechanisms are important for differential MVP MVIC mechanobiological phenotypes are therefore warranted. Several possibilities for uncoupling are likely, including reduced integrin expression [43], reduced integrin-ECM binding activity [44], reduced attachment of adhesions to the cytoskeleton via talin and vinculin [45], and reduction in organization of the deposited ECM, the intracellular cytoskeleton, or cellular morphology [46]. MVP MVICs displayed a shift in traction force dominance from expansile to contractile regions relative to normal cells, highlighting a potential role for altered cell morphology in the disease phenotype. In addition to actin and focal adhesion components, other cytoskeletal elements such as microtubules may also influence traction generation and intracellular strain, motivating further investigation into their role in MVP MVIC mechanobiology. These differences in cell-generated traction and polarity may ultimately influence how MVICs remodel and stiffen their surrounding ECM, motivating closer examination of local strain energy distributions.

Interestingly, the strain energy densities of the closest areas to the cell boundaries were actually higher in hydrogels modified by normal MVICs than in those modified by MVP MVICs, which then drops off more quickly in normal than in MVP samples. This suggests that MVP MVICs may have an impaired ability to mature the ECM proteins that they secrete. This aligns with what is seen in histology cross-sections of mitral valve leaflets, where MVP leaflets have a more unstructured ECM than normal leaflets [4]. Our future endeavors therefore involve further developing the inverse modeling pipeline for modulus determination so that anisotropic ECM modification can be elucidated in the analysis.

### Limitations

This study was able to determine the heterogeneous values of basal displacements, deformations, moduli, traction forces, and strain energy densities surrounding MVICs from normal and MVP patients. Due to experimental noise and the need to make the problem size tractable, only hydrogel displacements greater than 0.38 μm were reliably assessed. Although this represents very fine spatial resolution, some MVICs had to be excluded because the event horizon volume was too small. While this may have slightly affected the absolute average values, the observed trends between normal and MVP MVICs remain valid, as the same 0.38 μm cutoff was applied consistently across all analyses. When examining computed moduli, a liberal cutoff at the global median for stiffened or degraded regions was applied to minimize noise. Future work will aim to refine the cutoffs of 82 Pa and 36 Pa to values closer to the unmodified hydrogel modulus of 54 Pa. Nonetheless, the current methodology provides a rich quantitative understanding of ECM-rich hydrogel mechanobiology. Even small collagen deposits away from the cell body (likely laid down during cell migration [47, 48] were detectable implementing this workflow. Most important, our analysis demonstrates that MVP MVICs exhibit a paradoxical phenotype: they produce a stiffer ECM while imparting lower traction forces and strain energy densities onto that ECM.

Clearly, TFM remains a vibrant method for 3D analysis of the contractile and biosynthetic behaviors of cells. The present TFM computational pipeline allows for both more accurate and fuller analyses, due to its more realistic formulation, accounting for such problem features as regional varying moduli and gel compressibility. However, it is likely that the stiffer collagen deposition-rich regions may not be isotropic due to their fibrillar nature. We are thus currently upgrading our approach to use GPU based software coupled with quadratic elements for improved displacement and moduli gradient recovery. Moreover, we are exploring anisotropic material models for the gel to explore the possibility of spatially varying gel/collagen composite anisotropy.

### Conclusion

To summarize, while there are inherent limitations as in any study, our TFM modeling pipeline accurately captured key biological processes and reveal mechanical characteristics of MVIC remodeling behaviors that cannot be directly measured. Differences in biomechanical mechanisms that change in MVICs and their surrounding ECM during MVP were discerned. Through the implementation of 3D traction force microscopy [22, 23, 25] and our inverse modeling process [19], it was seen that MVP MVICs have larger basal contractile displacement and remodel their surroundings to become significantly stiffer. At the same time, MVP MVICs imparted significantly less traction force which led to significantly less strain energy density in the surrounding ECM-rich hydrogels of MVP MVICs. These results suggest that MVIC cellular phenotype (in normal and MVP MVICs) *intrinsically affected* basal contractility and ECM remodeling. Furthermore, the investigation’s results are in line with what is seen on an organ level during MVP, where leaflet thickening and stiffening, and ECM disorganization are hallmarks of the disease [4]. We also note that further investigation as to how MVP MVICs concurrently impart less traction and strain energy than normal cells even though they have higher expression of actin monomers including *α*SMA [4] is clearly warranted. Understanding this dichotomy that is seen here for the first time may be an important link in elucidating how MVP MVICs progress into their disease state. This will allow for future investigations on how to mitigate MVP progression biomechanically and how to improve primary surgical outcomes to prevent secondary procedures.

## Supplementary Material

supplementary material

**Supplementary Information** The online version contains supplementary material available at https://doi.org/10.1007/s10439-026-04319-y.

## Figures and Tables

**Table 1:** Two-tailed unpaired T tests with Welch’s correction results

Dependent variables	Norm mean ± SD	MVP mean ± SD	*p* value
Contractile displacement	1.266 ± 1.077 μm	1.565 ± 0.8742 μm	< 0.001
Stiffened moduli*	374.6 ± 803.9 Pa	445.7 ± 850.7 Pa	< 0.001
Degraded moduli*	16.93 ± 8.9556 Pa	19.66 ± 9.526 Pa	< 0.001
Unsigned surface traction	3.193 ± 14.94 kPa	0.9351 ± 0.7008 kPa	< 0.001
Event horizon strain energy density	0.9552 ± 3.449 Pa	0.7439 ± 2.339 Pa	< 0.001

* Stiffened moduli are defined as those greater than median stiffening of 82 Pa and degraded moduli are those less than median degradation of 36 Pa

**Table 2:** MVP MVICs exert significantly less unsigned traction in both contractile and expansile cellular regions

	Tractions (kPa)		
	Norm mean ± SD	MVP mean ±SD	*p* value*
Contractile	4.081 ± 27.02 kPa	2.148 ± 20.19 kPa	0.0145
Expansile	9.418 ± 44.01 kPa	0.3360 ± 2.496 kPa	< 0.001
*p* value*	< 0.001	0.7948	

* *p* values calculated between corresponding row or column comparison by two-way ANOVA with Tukey post-test

**Table 3:** Results for the strain energy density, which were higher in stiffened hydrogel zones and is lessened by MVP status

Zone	Norm mean ± SD	MVP mean ± SD	*p* value*
Stiffened	2.624 ± 6.086 Pa	1.615 ± 3.692 Pa	< 0.001
Degraded	0.3211 ± 1.306 Pa	0.2185 ± 0.3861 Pa	< 0.001
*p* value*	< 0.001	< 0.001	

* *p* values calculated between corresponding row or column comparison by two-way ANOVA with Tukey post-test

**Table 4:** Strain energy density is higher in hydrogel zones of contractile displacement and is lessened by MVP status

Zone	Norm mean ± SD	MVP mean ± SD	*p* value*
Contractile	1.377 ± 4.120 Pa	0.8854 ± 2.579 Pa	< 0.001
Expansile	0.4516 ± 2.317 Pa	0.4741 ± 1.764 Pa	0.2550
*p* value*	< 0.001	< 0.001	

* *p* values calculated between corresponding row or column comparison by two-way ANOVA with Tukey post-test

## Data Availability

MVICs were obtained from the Columbia Biobank for Translational Science. All other materials were commercially available. Data can be found on Mendeley at [49]. Code implemented for this study can be found at https://github.com/WCCMS-UTAustin/3DTFM
