# Supplementary material for: 3D Contractile and Remodeling Behaviors of Functionally Normal and Prolapsed Human Mitral Valve Interstitial Cells
Source: Ann Biomed Eng. Author manuscript; Available in PMC 2026 Sep 25. (PMC13614627; doi:10.1007/s10439-026-04319-y)
Supplement: Supplementary file 1 [file NIHMS2207815-supplement-supplementary_material.pdf]

Table 5 Stiffened Moduli by Distance Significantly Change by MVP Status

Stiffened Moduli			
**Distance from Cell Boundary**	**Normal Mean ± SD (Pa)**	**MVP Mean ± SD (Pa)**	**Tukey p Value***
0–10 μm	$2.249 \times 10^5 \pm 7.940 \times 10^6$	$1.500 \times 10^5 \pm 5.158 \times 10^6$	1.000
10–20 μm	$7.401 \times 10^4 \pm 3.345 \times 10^6$	$7.773 \times 10^2 \pm 6.343 \times 10^3$	1.000
20–30 μm	$1.962 \times 10^6 \pm 5.929 \times 10^7$	$7.244 \times 10^2 \pm 8.207 \times 10^3$	< 0.001
30–40 μm	$3.498 \times 10^2 \pm 5.556 \times 10^3$	$7.657 \times 10^2 \pm 7.824 \times 10^3$	1.000
40–50 μm	$1.070 \times 10^2 \pm 2.824 \times 10^1$	$8.943 \times 10^2 \pm 6.645 \times 10^3$	1.000
50–60 μm	$1.216 \times 10^2 \pm 4.089 \times 10^1$	$1.362 \times 10^2 \pm 8.675 \times 10^1$	1.000
60–70 μm	$1.247 \times 10^2 \pm 4.902 \times 10^1$	$1.409 \times 10^2 \pm 8.944 \times 10^1$	1.000
70–80 μm	$9.892 \times 10^1 \pm 1.681 \times 10^1$	$1.715 \times 10^2 \pm 1.627 \times 10^2$	1.000
80–90 μm	$1.072 \times 10^2 \pm 1.954 \times 10^1$	$1.322 \times 10^2 \pm 8.880 \times 10^1$	1.000
90–100 μm	$1.035 \times 10^2 \pm 1.348 \times 10^1$	$1.015 \times 10^2 \pm 8.321 \times 10^0$	1.000

*Tukey p values were calculated from the corresponding row comparisons with a two-way ANOVA and Tukey post-test. The two-way ANOVA also found that $p = 0.01166$ for a difference in MVP status and $p = 0.09257$ for a difference in distance.

Table 6 Degraded Moduli Significantly Change by MVP Status and Distance from the Cell Boundary

Degraded Moduli			
**Distance from Cell Boundary**	**Normal Mean ± SD (Pa)**	**MVP Mean ± SD (Pa)**	**Tukey p Value***
0–10 μm	18.56 ± 9.106	17.51 ± 10.28	0.1589
10–20 μm	21.20 ± 8.656	21.68 ± 9.392	0.9820
20–30 μm	19.51 ± 9.211	22.81 ± 9.094	< 0.001
30–40 μm	18.20 ± 9.373	21.17 ± 9.408	< 0.001
40–50 μm	17.24 ± 9.310	19.35 ± 10.00	< 0.001
50–60 μm	16.58 ± 9.128	19.15 ± 9.595	< 0.001
60–70 μm	16.07 ± 9.084	19.36 ± 8.819	< 0.001
70–80 μm	16.03 ± 8.937	23.79 ± 10.16	< 0.001
80–90 μm	15.94 ± 8.721	34.13 ± 0.6185	0.3416

*Tukey p values were calculated from the corresponding row comparisons with a two-way ANOVA and Tukey post-test. The two-way ANOVA also found that $p < 0.001$ for differences in both MVP status and distance.

5 Supplementary Information

Tables 5, 6, and 7 are presented below.

Table 7 Hydrogel Strain Energy Density Significantly Changes by MVP Status and Distance from the Cell Boundary

	Strain Energy Density		
**Distance from Cell Boundary**	**Normal Mean ± SD (Pa)**	**MVP Mean ± SD (Pa)**	**Tukey p Value***
0–10 μm	1.103 ± 2.034	1.364 ± 1.912	< 0.001
10–20 μm	0.5511 ± 1.324	0.4295 ± 0.9011	< 0.001
20–30 μm	0.1706 ± 0.5935	0.1387 ± 0.3611	< 0.001
30–40 μm	0.05540 ± 0.2991	0.05419 ± 0.1494	1.000
40–50 μm	0.02069 ± 0.08755	0.02113 ± 0.09472	1.000
50–60 μm	0.01219 ± 0.02726	0.01079 ± 0.02052	0.9987
60–70 μm	0.01027 ± 0.01987	0.01024 ± 0.01843	1.000
70–80 μm	0.009558 ± 0.01681	0.01024 ± 0.01843	1.000
80–90 μm	0.01082 ± 0.02022	0.01619 ± 0.03974	0.7366
90–100 μm	0.01564 ± 0.07038	0.01853 ± 0.06382	1.000

*Tukey p values were calculated from the corresponding row comparisons with a two-way ANOVA and Tukey post-test. The two-way ANOVA also found that $p < 0.001$ for differences in both MVP status and distance.
